# Optimizing song retention through the spacing effect

**DOI:** 10.1186/s41235-021-00345-7

**Published:** 2021-12-11

**Authors:** Joel J. Katz, Momo Ando, Melody Wiseheart

**Affiliations:** 1 Department of Music, York University, Toronto, ON Canada; 2 Department of Psychology, York University, Toronto, ON Canada; 3 LaMarsh Centre for Child and Youth Research, York University, Toronto, ON Canada

**Keywords:** Spacing effect, Distributed practice, Music, Song, Long-term memory

## Abstract

The spacing effect refers to the improvement in memory retention for materials learned in a series of sessions, as opposed to massing learning in a single session. It has been extensively studied in the domain of verbal learning using word lists. Less evidence is available for connected discourse or tasks requiring the complex coordination of verbal and other domains. In particular, the effect of spacing on the retention of words and music in song has yet to be determined. In this study, university students were taught an unaccompanied two-verse song based on traditional materials to a criterion of 95% correct memory for sung words. Subsequent training sessions were either massed or spaced by two days or one week and tested at a retention interval of three weeks. Performances were evaluated for number of correct and incorrect syllables, number of correctly and incorrectly pitched notes, degree notes were off-pitch, and number of hesitations while singing. The data revealed strong evidence for a spacing effect for song between the massed and spaced conditions at a retention interval of three weeks, and evidence of no difference between the two spaced conditions. These findings suggest that the ongoing cues offered by surface features in the song are strong enough to enable verbatim recall across spaced conditions, as long as the spacing interval reaches a critical threshold.

## Significance statement

The spacing effect is the finding that memory retention is improved when learning episodes are spaced out rather than massed into a single learning episode. To date, research has focused on rote memory for small pieces of information, such as vocabulary words. We examined whether the spacing effect improved song learning. There was a strong evidence of a benefit to memory for lyrics, but less convincing evidence for improved retention of the melody line. Unlike most studies, we failed to show benefits of further memory improvements with longer spacing between learning episodes. These results suggest that songs, which contain an abundance of retrieval cues, result in more robust and long-lasting memory formation.

## Introduction

Singing is one of the oldest means of transmitting long sections of text with remarkable stability (Rubin, 1995). Like other forms of music performance, it involves the unspooling of a long chain of association, where what is to come is cued by what is taking place (Chaffin et al., 2015). Song performance in particular requires continuous verbatim recall according to an imposed rhythmic and melodic pattern. As any singer will tell you, there is simply no time in singing to pause and search for the next word. In a song, the rhythm and prosody of the lyrics and the rhythmic and pitch constraints of the tune function as a framework for the song materials that constrain possible word and note choices (Rubin, 1995, 2006; Wallace & Rubin, 1991). This framework is presented at the first learning episode. When prosodic aspects of the poetic form—including stress patterns, rhyme, alliteration, and verse structure—are understood and the musical pitch and rhythm learned, the framework is in place. The words are then associated with the rhythmic, prosodic and melodic pattern through repetition (Rubin, 1995). Multiple cues combine to constrain the number of possible word choices in any given context. In an experiment testing 127 undergraduates for the effectiveness of rhyme and meaning used individually and then together as cues, researchers found that the probabilities of responding with the target words, given the rhyme, meaning, and dual cues, were 0.192, 0.142, and 0.973, respectively (Rubin & Wallace, 1989). The observed effect for dual cuing was three times the maximum predicted under existing models.

Even with this system of onboard constraints in song material, there are times when verbatim performance fails. Errors in vocal performance are usually failures to find the right word at the right time. Studies of expert piano and vocal performance (Chaffin & Imreh, 2002; Ginsborg & Chaffin, 2007) emphasize memory as content, which is addressable through declarative performance cues. The performer can continue from any point of hesitation by using conscious declarative information as cues. These cues are associated with a particular position in the musical structure during practice. An example might be, “Okay, here comes that spot where you have to go back to the opening.” Any singer will tell you that most of the time they sing from the feeling—not thinking of what is to come, but rather, experiencing the life of the song as it unfolds. As such, a singer’s experience is closer to that of an actor, who remembers text through the constraints of motivation, situation, character, and the intention to communicate (Noice & Noice, 1999). Unlike actors, singers are further constrained by the rhythmic structure of the music to retrieve words without hesitation. They must optimize memory security in performance without conscious self-cuing.

One of the most studied strategies for enhancing memory performance is the spacing effect (Carpenter et al., 2012; Pashler et al., 2007; Wiseheart et al., 2019). The spacing effect refers to an observable boost to memory performance when learning is distributed over a number of different sessions (spaced learning) compared to learning in a single session (massed learning). In spacing studies, the break between the learning sessions, or inter-study interval (ISI), may be minutes, days, weeks, or even months later (Cepeda et al., 2008, 2009). The time between the final learning session and the final test is called the retention interval (RI), which may vary widely. In a typical spacing study, there are two learning events and a final test. The first session presents the material for learning. If participants learn to a criterion (e.g., 95% correct) during session 1, researchers can ensure that all participants adequately learned the material. The second session usually involves relearning using a fixed number of relearning trials, which avoids a confound between ISI and amount of relearning. Otherwise, less well remembered material might be rehearsed a greater amount. The RI is typically fixed, which allows the ISI to be examined as a single independent variable.

Two major theories exist to explain the spacing effect. Encoding variability suggests that multiple cues are stored with learned items (Glenberg, 1979). When two or more learning episodes are spaced over time, a broader range of unconscious contextual cues are encoded that may then overlap with the context-dependent cues available at testing (contextual variability). More available cues mean more likelihood of retrieval. Eventually, the change in time between study sessions reaches a certain optimal point for any given retention interval. Beyond that optimal point, an increase in contextual elements is overtaken by the drift in context at time of testing away from the context of the learning sessions (Lindsey et al., 2009; Mozer et al., 2009). With drift, the context at testing shares fewer contextual cues with the learning sessions, and the material is less likely to be retrieved.

Study-phase retrieval suggests that a difficult retrieval at the second learning event will result in formation of more robust representations than an easy retrieval (Delaney et al., 2010; Thios & D’Agostino, 1976). With short inter-study intervals, materials studied at the first session are easily retrieved. As the spacing interval increases, the learned material is partially forgotten and must be reconstructed to be remembered. The extra effort required to reconstruct the memory is often termed “desirable difficulty” (Bjork, 1994). The memory trace of the item will be strengthened as long as an opportunity is given through restudy to correct any lapses in production. A corollary to this theory is that if too much time elapses between the study events, the item may be forgotten entirely. In this case, there is no strengthening of the initial memory trace; the learning material is instead encoded as a new event, and there will be no study-phase retrieval.

Song memory involves verbatim recall of poetic passages (lyrics) coupled with accurate recall of pitch sequences. Following the classification used in Donovan and Radosevich (1999), it is an example of high mental requirements coupled with high overall complexity and high physical requirements. No song spacing studies exist in the literature. However, a significant body of work exists on long-term memory for songs in the oral or ballad tradition. Wallace and Rubin (1988b) examined constraints within ballads for their effect on recall in a population of non-specialists. Twenty-seven undergraduates listened to ten repetitions of an unfamiliar ballad and were tested for word retention (in writing) after ten minutes. Imagery, metrical agreement, and causal connectedness all correlated significantly with recall—all features that had been observed in expert ballad performers (Wallace & Rubin, 1988a). Furthermore, when pairs of words in the same ballad were changed so that instances of assonance and alliteration were removed, significantly fewer of the changed words were recalled. Finally, where spoken recitation was heard, those lines which corresponded most closely to the overall metrical pattern were remembered best in a rhythmic recitation, a result consistent with rhythmic information acting to cue word recall. (Wallace & Rubin, 1988b). The different constraints can be regarded as schemas, not only for meaning, but also for poetics, rhythm, imagery, and music.

In an experiment testing 127 undergraduates for the effectiveness of rhyme and meaning used individually and then together as cues, Rubin and Wallace (1989) found that the probabilities of responding with the target words, given the rhyme, meaning, and dual cues, were 0.192, 0.142, and 0.973, respectively. The observed effect for dual cuing was three times the maximum predicted under existing models. A specific example taken from Rubin and Wallace is illuminating. The linguistic/semantic cue “building material,” for example, cued the word “steel” with a probability of 0.00; the auditory cue “rhymes with eel” also cued the target word with a probability of 0.00. The combined cue, “a building material that rhymes with eel,” cued the target with a probability of 1.00 without prior learning, even though the expected probability of the cue being effective was 0.00 (using the formula pa + pb − [pa x pb]) (Rubin, 2006). Based on the characteristics of the ballad form, and a certain amount of experimental evidence as cited above, Rubin found that in the ballad form at least, recall is serial; what is sung cues what is to come. Ongoing cues are based on poetic devices, including rhyme, alliteration, and assonance; meaning, visual imagery, and spatial imagery, which also function in a local, serial fashion; and rhythm, the only ongoing cue of a global associative nature (Rubin, 2006). Rhythm functions through repetition of a near-identical rhythmic pattern repeated throughout the verses. Multiple cues combine to constrain the number of possible word choices in any given context.

Although there are no spacing studies of poetry per se, there is a fair amount of research indicating that poetry offers a memory advantage over a comparable prose setting. Ebbinghaus (1885/1964) found that learning six stanzas of poetry took on average one-tenth the time of learning a comparable number of nonsense syllables. Using poetic and rhetorical materials, Rubin (1977) found that university undergraduates remember long stretches of five familiar texts (“The Preamble to the Constitution,” “The 23rd Psalm: A Psalm of David,” “Hamlet's Soliloquy,” “The Gettysburg Address,” and “The Star-spangled Banner”) verbatim, through associative chaining of surface elements. They showed no evidence of remembering in an abstract, reconstructive manner. Furthermore, recall was accurate and organized in terms of surface structure units. The prose materials in Rubin’s study share certain features with poetry, which could help to account for their memorability. They are rhetorical pieces written to be delivered in public address. They all have rhythmic patterning (not always regular), alliterative devices, and phrasing divided by points to take breath. They were often learned by memory through frequent exposure in early life, and all have important emotional resonance for American students.

While recognition memory for specific words in prose, separated by intervening text, diminishes greatly over short retention intervals (Sachs, 1967), recall of phrases in lyric poetry is not diminished (Tillman & Dowling, 2007). Moreover, verbatim memory for surface features of target syllables in poetry is better than for target syllables in prose. The authors suggest that both music and poetry offer semantic structures that facilitate recall of surface features based on rhythmic structure and temporal organization. Alliterative lines of poetry are more likely to be falsely recognized in both immediate recall and after 12 h compared to non-alliterative lines or paraphrases, indicating that alliteration as a formal, schematic device is preserved in memory and helps to cue memory (Atchley & Hare, 2013). Alliterative cues reactivate memory of previous information that is phonologically similar, effects holding for both poetry and prose (Lea et al., 2008). A continuous reading paradigm was used, so the effect of retention interval was not tested. Undergraduates will select words to complete sentences based both on rhyme and on meaning, supporting the importance of surface features in determining word choice (Rapp & Samuel, 2002).

A few studies have explored spacing effects for connected discourse (i.e., prose). Spacing effects have been shown for gist recall and comprehension of prose passages over retention intervals of up to two days (Glover & Corkill, 1987; Greving & Richter, 2019; Krug et al., 1990; Rawson, 2012; Rawson & Kintsch, 2005; Verkoeijen et al., 2008). These studies involved rereading, and none taught the material to a uniform criterion at the first session. Verkoeijen et al. (2008) tested free recall for connected discourse, combining verbatim, gist, and idea-unit memory. They used longer ISIs than the others (massed, 4-day, and 25-day ISIs) and an RI of two days. Retrieval improved between the massed and 4-day ISIs and declined for the 25-day ISIs, in keeping with the spacing literature for simple verbal materials. Because of the confound in variables measured, it is impossible to determine the effect of spacing on verbatim memory alone. Further studies are needed to establish spacing effects in verbatim retrieval of connected discourse over a range of different RIs.

Music spacing studies are usually confined to simple musical materials over short RIs. Using a short left-hand piano figure learned and tested from score, Simmons (2012) found fewer performance errors after an RI of 24 h for an ISI of 24 h compared with a shorter ISI of six hours. However, the study did not train participants to a uniform criterion of errors in the first session, making it impossible to separate the effects of differential learning from the effect of ISI. In addition, experimental results were reported as an average over multiple sessions, without a retention interval. Under these circumstances, benefits from spacing could not be determined. Rubin-Rabson (1940) evaluated learning of short piano pieces among experienced pianists. The methodology allowed a variable number of trials at the second learning session, so the effect of lag (gap between ISIs) was confounded by number of relearning trials. Cash (2009) studied the effect of a 5-min break on learning a keypress sequence or a sequence of 13 notes. Results showed improved performance for an early 5-min gap over a later 5-min gap when tested 12 h later after sleep. A study by Wiseheart et al. (2017) used five different ISIs between zero and 15 min and found no spacing effect at an RI of five min, either for piano keypresses with visual directions or memorized song fragments. The data indicated that no forgetting had taken place before the second learning session, hence study-phase retrieval could not occur. Studies by Stambaugh (2011) and Stambaugh and Demorest (2010) examined short phrases played on clarinet or saxophone for accuracy and musicality in a massed or interleaved (spaced) condition. These were not memory studies and did not use a lag between study events, limiting their applicability to this research.

### Current study

The current study is the first to evaluate the effect of spacing on song memory. Our theoretical perspective combines the theory of multiple constraints in song memory with encoding variability and study-phase retrieval, from the spacing effect literature. Remembering a song requires retrieval of the episodic traces representing exposure to the song in the learning phase of the experiment (Glenberg, 1979). Access to these traces is provided by the cue at testing. The cue allows for activation of components in the episodic trace identical to those in the cue (Lockhart, 2002). A song, such as a folk song or ballad based on traditional materials, may introduce cues related to multiple episodic systems (Rubin, 2006). This type of song contains: a wealth of associative elements related to ongoing constraints from the prosodic structure, imagery, rhyme, and meter of the text; the metrical and pitch characteristics of the musical setting; the narrative and affective nature of sung material; and the proprioceptive aspects of singing (Rubin & Wallace, 1989; Rubin et al., 1993; Wallace & Rubin, 1991). Structural components that are created by the poetic and musical framework are associated with the initial words and notes (the cue) during the learning phase, and then may be activated by the cue at testing. Thus, once the melody and words are learned together, retrieval of one enables retrieval of the other (Ginsborg & Sloboda, 2007).

To the extent that access to song memory is analogous to memory for words, access to the memory is predominantly controlled by the most specific components in the trace (Glenberg, 1979). Since structural components are more specific than contextual components, access to song memory should be primarily controlled by the structural components implied by the initial cue, and the ongoing associative cues generated by the performance as it unfolds.

Once sufficient structural information can be recovered from the cue, the words and notes of the original song can be reconstructed. In the massed condition, study-phase retrieval is too easy to allow strengthening of the initial memory in the restudy session; the learning context will not offer the variety of contextual cues that are available in the spaced conditions. Under these circumstances, after a medium to long retention interval structural components are less likely to be recovered from the cue and the song will be difficult to remember. When the structural components which allow retrieval of the song (e.g., the rhythmic pattern, the rhyme scheme, the metrical pattern of the melody, the narrative structure) are sufficiently associated with the cue to allow a reconstruction of the material, the song will be remembered. This will create a spacing effect when massed and spaced conditions are compared. Any boost to the memory trace offered by increased contextual variability and more difficult study-phase retrieval at longer spacing intervals will be overtaken by the structural components available from the cue. Under these circumstances, there will be no difference in recall at final testing between the two spaced conditions at the same retention interval.

After one learning episode, the structure of the song is only weakly associated with the cue. In the massed condition, the recency of the first session allows for retrieval of the song pattern (and hence the words and notes) at the second session. In the spaced conditions, the material is forgotten and the song structure and word and note associations will only be sufficiently strong to allow weak retrieval using structural cues. In both spaced conditions the normal forgetting curve, which is governed by contextual cues, will be overtaken by whatever structural cues recoverable from a single learning session. As a consequence, there will be no difference in forgetting for notes or words between the two spaced conditions.

## Method

### Participants

Our aim was to obtain a sample size that would allow us to find at least moderate evidence for either the experimental or null hypothesis for all major analyses, using Bayesian analyses. We estimated that *n* = 90 would be sufficient for this goal, based on an estimate of *d* = 0.85 in the verbal spacing effect literature (Cepeda et al., 2006). A total of 112 participants began the experiment. University students enrolled in the fall term (*n* = 91) were drawn from a second-year music skills class for music majors and received course credit for participation in the study. A further group in the spring term (*n* = 21) were recruited by poster from the general university community and given a coffee card as incentive. Twelve participants were excluded from all analyses for being above the cut-off age of 33 years old, having learning disabilities, or missing or overhearing sessions. A further 13 were unable to reach a criterion of 95% correct syllable retrieval in the first session and did not complete the study. The remaining 87 participants were on average 21 years old (*SD* = 3, range = 18–33), 45 were female and 42 were male, and 97% were native English speakers or had spoken English for 10 years or more. Guardians had 15 years of education, on average. Participants were Caucasian (*n* = 44), Asian (*n* = 26), Black (*n* = 12), or Hispanic (*n* = 5). Bayesian ANOVA or Bayesian multinomial tests (as appropriate) indicated no difference between groups for individual demographics (“Appendix 1, Table 5”).

Although testing was conducted by the lead researcher, who was not blind to condition, efforts were made to ensure freedom from bias and to establish equivalence between groups other than for the experimental manipulation. Of the three sessions in the lab, sessions 2 and 3 followed a strict protocol determined by the slide presentation. The initial session necessitated individualized coaching, so that participants would reach the criterion learning goal. The individual first sessions were compared post hoc to determine relative equivalence in coaching styles between groups. Forty-two participants out of the 87 who reached criterion were prompted to a verbal recitation of the song text. Analyses showed no difference between groups for time spent in verbal recitation (BF_10_ = 0.15). Some variation was found in vibrato, portamento, quality, pitch, and rhythm in the stimulus recordings depending on the personal characteristics of the singers and the degree of post-production editing by the researcher. Bayesian frequency analysis indicated no evidence of difference in distribution of stimulus materials across experimental conditions (BF_10_ = 1.98). At the end of the first learning session there was evidence of no difference between groups for words or notes on any of the measured parameters. Overall, the song was learned to the same (correct) standard across groups (“Appendix 2, Tables 7 and 8”).

### Design

Participants were given an initial study session where they were trained to a criterion of 95% correct word recall, followed by a review session after 10 min (massed) or 2 days or 1 week later (spaced), and then a final test three weeks after the review session (“Appendix 3”). Participants were randomly assigned to the 10-min (massed), spaced at two days, or spaced at 1 week training conditions. Final tests were cued first by notes alone and then by notes and words. Word (i.e., syllable) accuracy and note accuracy (i.e., number of correctly sung notes and absolute value of deviation in cents from the pitch of the stimulus materials) at the final test were the dependent variables for hypotheses 1 and 2. Word and note accuracy at the start of session 2 were the dependent variables for hypotheses 3 and 4. There were 30 participants in the 10-min (massed) group, 28 with a 2-day ISI and 29 with a one-week ISI.

### Materials

A song was newly composed by the first author, based on “Come all ye old comrades,” song 59 of the Traditional Songs from Nova Scotia (Creighton & Senior, 1950). Efforts were made to respect and enhance the melodic simplicity, rhythmic regularity, consistent rhyming structure, and concrete textual imagery characteristic of songs in the oral tradition (Wallace, 1994). A text was composed that respected the rhythmic profile of the original (“Appendix 4”). The newly composed words had the following syllable count: Verse 1: 11/12/12/12 syllables; Verse 2: 11/12/12/12 syllables (total of 94 syllables for the song). The lyrics had a Flesch Kincaid Grade Level of 0.7 (Kincaid et al., 1975), a Flesch Reading Ease score of 100.00 (Flesch, 1948), and a Gunning Fog index of 4.3 (Gunning, 1971), indicating an extremely easy read.

The song scores were prepared using Noteflight, an online music transcription software, with notation in the treble clef in the keys of F for soprano, E-flat (Eb) for mezzo, and D for alto voice, in the tenor treble clef in the key of F for tenor, and in the bass clef in D for baritones and C for basses. The songs were recorded on the piano in the appropriate key and octave for the different voice types by a pianist in a professional performance program at a local conservatory, using a click track set to quarter note = 138, a tempo chosen to sound natural with the words. The songs were then recorded by three singers (undergraduates in a professional vocal program) using a click track set to quarter note = 138, with a Steinberg microphone and preamplifier in Logic Pro software using a MacBook computer. The soprano recorded the material twice, once in F and once in Eb. The Eb version was transposed into D to produce the alto materials using Melodyne, a professional note-editing program. The tenor version (in F) was recorded separately, as was the baritone (in D). The baritone performance was transposed one tone down into C to generate bass materials. Six different PowerPoint presentations in the different stimulus keys were then prepared using the stimulus recordings. A script was written for each of the three sessions, with the stimulus recordings embedded in the presentation. The researcher then recorded instructions and prepared instructional slides to go with the recordings.

### Procedure

The participants were first exposed to the two-verse song melody played on the piano in a key appropriate to their voice type. After the song melody was presented and imitated in line-by-line, phrase-by-phrase, and complete form, participants attempted to sing the song from score. Learning trials then continued until a maximum of four tests had been given, or until the participant reached the criterion of a correct performance of the melody, as judged by the lead researcher, a professional singer and music teacher with over 40 years of experience in the field. All participants were then exposed to the learning trials for the song with words, whether the tune had been correctly learned or not. Presentation of the song proceeded similarly to the presentation of the tune, this time using the vocal recordings and the score with words. The song with words was presented and imitated in line-by-line, phrase-by-phrase, and complete form, until participants were ready to try the song from memory. Those who indicated they were not ready were then coached by the researcher so that they might reach criterion within the allotted maximum session time of 45 min. All participants continued with learning trials and memory testing until criterion was reached, or the 45 min allotted for the session had elapsed. At the end of first session testing, a demographic questionnaire was completed. Massed (10-min ISI) participants were engaged in conversation for the remainder of the 10-min between sessions to prevent active rehearsal of the materials. Participants in the spaced conditions were thanked for their participation, reminded of the second appointment and asked not to practice the material or otherwise think about the song between sessions.

The second session procedure was uniform across all conditions. Participants were given two initial memory tests, the first cued by first notes, then second by first notes and words. Participants were exposed three times to the stimulus materials and instructed by slide to sing along. They were then given the final memory tests for the second session, thanked, reminded of their final appointment in three weeks, and asked not to review or otherwise think about the studied material. At the third testing session, tests with note cue and note and word cue were given. Although sung performance of text was requested, credit for any correct words spoken in the rhythm of the poem was also granted. Any notes sung without words were also included. Participants who did not reach criterion in the final test were given further training with the recording and score and tested after each training until they reached criterion for the words. None of the participants, when queried, admitted to conscious review of the material. All participants in the study were then given the Mini-PROMS test (Profile of Music Perception Skills; Zentner & Strauss, 2017), a well-validated 15-min version of the original PROMS battery of tests (test–retest reliability, *r* = 0.83. Criterion validity, *r* = 0.61). Results indicated no difference between groups for musical perceptual ability (BF_10_ = 0.17).

### Data coding and analyses

Session files for each participant were converted into a blinded format by one of the authors not involved in testing. Anonymized files were then downloaded into Melodyne, an audio processing software. Melodyne uses a fast Fourier transform (FFT) to separate the test files into separate notes according to its proprietary algorithm (Neubäcker, 2009). Ten percent of the note assigned files were checked by a second rater. Agreement between raters for word and note omissions ranged from 0.986 to 1.0 (Pearson’s *r*; “Appendix 1, Table 6”). Once the notes were assigned in the Melodyne files to the satisfaction of the lead researcher, the algorithmically generated values for pitch (in note names and cent deviations) and note length, breaths, and hesitations (in hundredths of a second) were then transcribed by one of three different coders and entered into spreadsheets.

Data collected during the sessions allowed examination of word and note memory and note accuracy. Time to learn during the first session, time to relearn in the third session, and number of relearning trials to reach criterion in the third session were also tracked. Word memory included number of correct syllables, number of syllable additions, and number of incorrect syllables recalled. There were almost no syllable or note additions, so those data were not reported. Note data included number of on-pitch quarter notes (no more than 50 cents off pitch), number of off-pitch quarter notes (more than 50 cents off pitch), and absolute value of cents off-pitch for quarter notes. We also included number of hesitations while singing (defined as text repetitions outside the parameters of the song or pauses added by the singer). Results were analyzed with Bayesian ANOVAs and post hoc tests for unimodal data (number of correct syllables [session 1], number of incorrect syllables, number of correctly and incorrectly pitched quarter notes, absolute value of cents off pitch, number of hesitations, and time and trials to reach criterion) and Bayesian multinomial tests for bimodal data (number of correct syllables and notes [sessions 2 and 3]). For multinomial tests, number of correct syllables was split as 1–46 or 47–92 syllables correct, and number of notes correct was split as 1–36 or 37–72 notes correct. Data and materials are available at https://osf.io/mus3c/.

While there were 94 syllables in the song, for analysis, syllables were computed out of 92; the first two syllables were used as cues and thus discarded for analysis. There were 74 quarter notes in the song, but the first two were cues, and thus were discarded, with a maximum possible of 72 quarter notes correctly sung. We did not analyze eighth notes (which occurred at the beginning of phrases), because they are frequently sung slightly off pitch, as passing notes. Nor did we analyze dotted quarter or half notes, which occurred at the end of phrases in the verses.

## Results

**Fig. 1:**
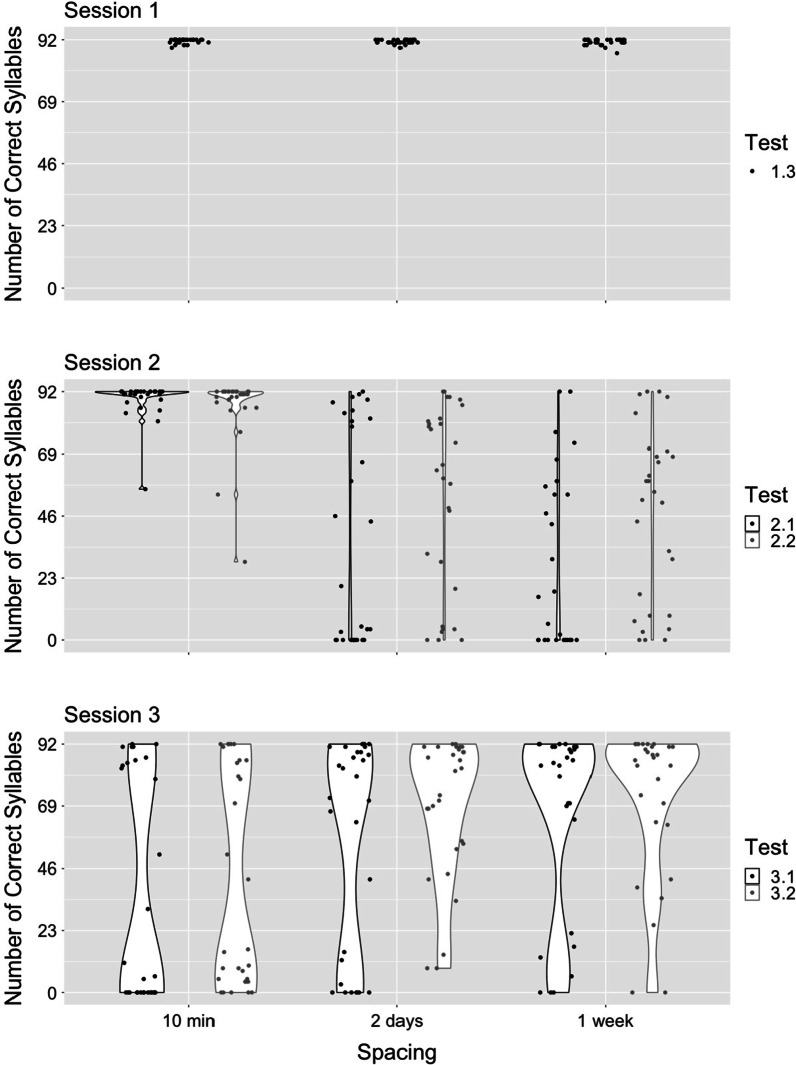
Number of correct syllables (test 1: cued by notes; test 2: cued by notes and words)

**Fig. 2:**
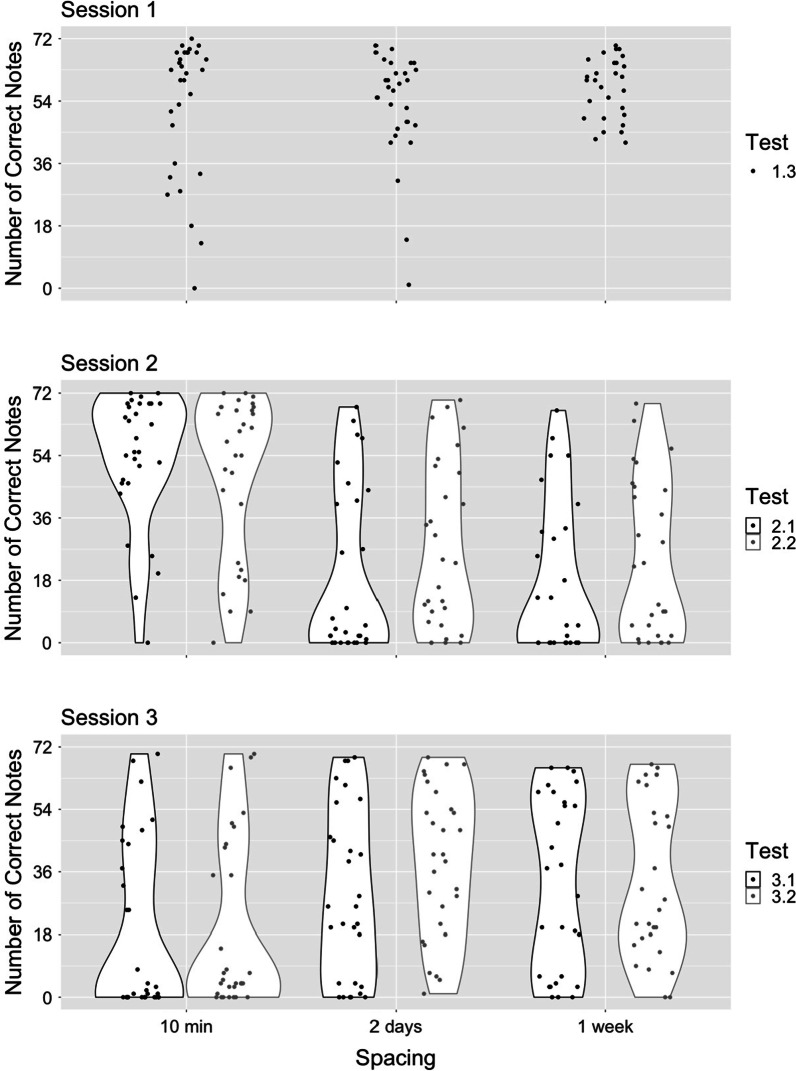
Number of correct notes (test 1: cued by notes; test 2: cued by notes and words)

We examined forgetting, using two tests at the beginning of session 2 (without initial note cues [test 2.1], followed by with initial note cues [test 2.2]). We expected that the massed group would show an advantage for syllable and note retrieval, and that there would be no difference for syllable or note retrieval between the 2-day and 1-week spaced conditions. Results confirmed our predictions (Figs. 1 and 2; Tables 1 and 2). At both tests, participants in the massed condition recalled more correct syllables than those in the spaced conditions, and there was no difference between participants in the 2-day and 1-week condition. The same pattern held for both correct pitch recall and absolute value of cents off pitch for quarter notes; participants in the massed conditions recalled notes better than those in the spaced conditions, which did not differ. Evidence supports the conclusion that after the initial session, participants forgot most of the song (both notes and words) in both spaced conditions but remembered it almost perfectly in the massed condition.

**Table 1:** Measures of forgetting at test 2.1

Measure	Massed *M* (*SD*)	2 day M (*SD*)	1 week M (*SD*)	BF_10_ Overall	Posterior odds massed vs. 2 day	Posterior odds massed vs. 1 week	Posterior odds 2 day vs. 1 week
Number of correct syllables (ANOVA)	89.2 (6.7)	38.3 (39.7)	30.2 (32.6)	5.4 × 10^8^	1.2 × 10^6^	1.9 × 10^10^	0.21
Proportion of participants who correctly remembered half the lyrics (multinomial)	1.00	.41	0.39	4.9 × 10^6^	3.4 × 10^5^	6.0 × 10^5^	0.32
Number of incorrect syllables	1.1 (1.8)	2.4 (3.9)	2.5 (4.0)	0.37	0.52	0.54	0.16
Number of correctly pitched quarter notes (ANOVA)	53.4 (19.1)	19.5 (24.2)	17.8 (22.1)	3.5 × 10^6^	4.4 × 10^4^	3.5 × 10^5^	0.16
Proportion of participants who correctly sang half the notes (multinomial)	0.83	0.31	0.21	6.1 × 10^5^	1.4 × 10^3^	3.2 × 10^4^	0.39
Number of incorrectly pitched quarter notes	17.2 (18.0)	17.7 (21.1)	14.8 (19.0)	0.12	0.16	0.17	0.18
Absolute value of cents off pitch quarter notes	36.6 (23.3)	189 (232)	106 (115)	24.8	22.3	8.6	0.38
Number of hesitations	1.9 (2.1)	1.7 (2.4)	2.5 (6.0)	0.13	0.16	0.18	0.19

**Table 2:** Measures of forgetting at test 2.2

Measure	Massed *M* (*SD*)	2 day M (*SD*)	1 week M (*SD*)	BF_10_ Overall	Posterior odds massed vs. 2 day	Posterior odds massed vs. 1 week	Posterior odds 2 day vs. 1 week
Number of correct syllables (ANOVA)	86.8 (13.1)	52.8 (34.6)	45.9 (32.4)	3.7 × 10^4^	1.7 × 10^3^	1.6 × 10^5^	0.20
Proportion of participants who correctly remembered half the lyrics (multinomial)	0.97	0.66	0.57	115	30.0	227	0.38
Number of incorrect syllables	1.8 (2.5)	4.3 (5.1)	3.6 (4.5)	0.92	1.6	0.67	0.18
Number of correctly pitched quarter notes (ANOVA)	49.1 (23.2)	27.2 (23.9)	23.8 (22.9)	233	23.8	127	0.18
Proportion of participants who correctly sang half the notes (multinomial)	0.73	0.34	0.36	26.6	26.6	18.9	0.31
Number of incorrectly pitched quarter notes	21.3 (21.1)	20.6 (19.4)	20.4 (18.0)	0.10	0.16	0.16	0.16
Absolute value of cents off pitch quarter notes	45.4 (35.0)	99.5 (101)	116 (126)	3.5	3.2	5.2	0.19
Number of hesitations	1.6 (2.3)	2.1 (3.1)	2.0 (2.4)	0.14	0.20	0.20	0.16

We ran mixed-measures ANOVAs with session (session 1 [test 1.3] and session 2 [test 2.2]) and spacing (10-min, 2-day, and 1-week) as factors. Participants made more syllable errors at the start of the second session compared to the end of the first session, BF_10_ = 7.0 × 10^5^. It is inconclusive whether the increase in syllable error rate differed between groups, BF_10_ = 0.98. It is inconclusive whether participants made more note errors at the start of session 2, BF_10_ = 0.71, and there is evidence against an interaction between session and spacing, BF_10_ = 0.17. Participants were more off pitch at the start of session 2, BF_10_ = 1.8 × 10^3^. Participants in the spaced conditions showed a greater increase in pitch errors, shown by an interaction between session and spacing, BF_10_ = 10.6.

### Final test

Our main goal was to study the effect of three different spacing intervals on final test performance of a song after a three-week retention interval. Results bore out our predictions for word learning but not for note learning (Tables 3 and 4). There was a clear benefit to word learning, measured by number of correct syllables, at test 3.2, and no difference between 2-day and 1-week gap performance. Thus, we found support for hypotheses 1 and 2, for word learning. There was only a benefit between massed and 1-week spacing intervals at test 3.1, perhaps because when cued only by notes, it was quite challenging to remember the words.

**Table 3:** Spacing effects at test 3.1

Measure	Massed *M* (*SD*)	2 day M (*SD*)	1 week M (*SD*)	BF_10_ Overall	Posterior odds massed vs. 2 day	Posterior odds massed vs. 1 week	Posterior odds 2 day vs. 1 week
Number of correct syllables (ANOVA)	35.5 (41.6)	54.5 (39.6)	65.6 (34.8)	3.4	0.59	5.5	0.27
Proportion of participants who correctly remembered half the lyrics (multinomial)	0.40	0.62	0.75	3.4	1.3	11.2	0.51
Number of incorrect syllables	1.2 (2.9)	3.1 (4.6)	2.6 (3.5)	0.58	0.75	0.56	0.17
Number of correctly pitched quarter notes (ANOVA)	19.2 (24.4)	29.2 (24.4)	32.3 (25.2)	0.60	0.44	0.81	0.17
Proportion of participants who correctly sang half the notes (multinomial)	0.30	0.41	0.50	0.28	0.45	1.0	0.39
Number of incorrectly pitched quarter notes	12.4 (17.1)	21.3 (20.7)	23.3 (20.9)	0.75	0.59	1.1	0.17
Absolute value of cents off pitch quarter notes	113 (162)	61.2 (46.4)	69.1 (50.8)	0.44	0.41	0.34	0.19
Number of hesitations	0.67 (1.2)	2.1 (3.0)	1.3 (1.6)	1.4	1.5	.44	0.31

**Table 4:** Spacing effects at test 3.2

Measure	Massed *M* (*SD*)	2 Day *M* (*SD*)	1 Week *M* (*SD*)	BF_10_ overall	Posterior odds massed vs. 2 day	Posterior odds massed vs. 1 week	Posterior odds 2 day vs. 1 week
Number of correct syllables (ANOVA)	40.6 (39.8)	68.9 (26.4)	71.8 (28.2)	64.3	9.4	15.7	0.17
Proportion of participants who correctly remembered half the lyrics (multinomial)	0.43	0.79	0.79	15.4	16.4	12.8	0.26
Number of incorrect syllables	1.9 (3.8)	3.8 (4.6)	2.9 (2.9)	0.40	0.51	0.25	0.22
Number of correctly pitched quarter notes (ANOVA)	19.4 (24.3)	38.7 (21.2)	33.6 (22.8)	8.2	10.1	1.4	0.21
Proportion of participants who correctly sang half the notes (multinomial)	0.26	0.55	0.42	0.99	3.5	0.68	0.49
Number of incorrectly pitched quarter notes	17.2 (20.8)	23.6 (16.4)	27.3 (20.4)	0.50	0.32	0.64	0.20
Absolute value of cents off pitch quarter notes	93.4 (98.8)	82.6 (125)	67.1 (49.3)	0.16	0.17	0.30	0.19
Number of hesitations	1.0 (1.2)	1.4 (2.4)	1.3 (1.9)	0.14	0.21	0.20	0.16

While means suggested that the massed group performed worse than the spaced groups at note learning, the only statistically conclusive analysis showed a benefit for 2-day spaced vs. massed ISIs, at test 3.2. Thus, we have equivocal support for hypothesis 1, for note learning. We found clear evidence in support of hypothesis 2, for note learning, with Bayes factors that supported a null difference between the spaced groups.

Time and trials to relearn the song were approximately the same between groups (“Appendix 2, Table 9”). There was equivocal evidence for syllable and note errors and number of hesitations.

## Discussion

The findings of the present study suggest that spaced practice is an effective means of enhancing song retention. Extending the lag between spaced repetitions of a song from two days to one week does not show the improvement in memory scores that might be expected from comparable materials in studies of verbal learning (Cepeda et al., 2006). The present study is the first spacing study of song materials and the first to demonstrate that spacing song learning enhances retrieval. This finding has implications for cognitive theories of song retrieval and practical implications for the effective performance of songs from memory.

Ginsborg and Chaffin (2007), in their study of the preparation of a movement from the Stravinsky Ricercar for performance by one of the authors, singer Jayne Ginsborg, found through content analysis that performance cues were important in establishing memory security. These conscious declarative cues are linked to specific places in the musical score and were originally noted in the preparation of pianist Gabriel Imreh for a performance of the third movement of Bach’s Italian Concerto. Our study differs from both of these studies in several important respects. Both the Ricercar and the third movement of Bach’s Italian Concerto are complex works, which may demand conscious declarative cuing to be retrieved accurately in performance. We examined amateur singers learning a simple song based on the ballad tradition and introduced an experimental manipulation (spacing of study episodes) that varied the unconscious cues available at the time of retrieval. We found that spacing learning sessions made a great difference in the amount of material remembered by the participants. Our findings support a theory of song memory where intuitive learning cues memory of specific features in the song based on structural constraints in the materials (Rubin, 1995).

When structural cues prompt ongoing retrieval, results will tend toward an all-or-nothing response. For retrieval, the song structure must be recovered from the initial cue. Once this happens, the ongoing unspooling of the song will continue, provided that a critical interval for learning consolidation has been reached. Without this learning consolidation, the material is insufficiently associated with the structural cues. After one learning session, the song pattern is only weakly associated with either the note cue or the note and word cue. Most participants will not recover this pattern from the cue. In a few cases, however, it will be recovered and the song will be largely remembered. This conclusion is borne out by the data. At the first tests of session two, the song was completely remembered by most of the participants in the massed condition. At both two-day and one-week gaps, there was a bi-modal distribution of scores ranging from mostly forgotten to mostly retrieved. At the final tests, retrieval was low in the massed condition and high at both spaced intervals, also with a bi-modal distribution. In all cases, a few participants remembered the song accurately. If contextual variability had been the primary driver of recall, we would have expect increased forgetting from the two-day to the one-week interval, as the match between learning context and testing context diminished, and a difference between the two spaced conditions at final testing.

At present, no mathematical model exists for the interplay of structural and contextual cues in the recovery of complex materials. Such a model would enable our predictions to be more solidly grounded in theory. It is possible to sketch out what such a model could look like, based primarily on Glenberg’s (1979) model. Encoding variability presents a hierarchy of cue types that govern retrieval of verbal materials, from the most specific (descriptive cues) to the least specific (contextual cues), with structural cues operating in between. Memory retrieval is governed by the most specific cues in this hierarchy. Glenberg’s mathematical model deals only with the summative operation of contextual cues. A model for song memory would allow for the simultaneous operation of descriptive performance cues, structural cues related to the poetic and musical constraints in the song, and contextual cues offered by the learning and testing environments. A complete description of the song through performance cues is an impossible burden for the performer. Their use would be limited to moments in the song that are vulnerable to forgetting. The total pattern of possible structural cues is fixed by the stimulus materials (the song), and their contribution depends on how much of the structure has been recovered. Contextual cues should always contribute, to some degree, but their weighted strength should depend on the degree to which structural cues have been retrieved and the extent to which performance cues have been explicitly added to the rehearsal process. A more exact description of the interplay between descriptive, structural, and contextual cues in song retrieval awaits further experimental testing.

There was inconsistent evidence for a spacing effect for sung notes. Only one of four comparisons showed support for a spacing effect (test 3.2, 10-min vs. 2-day ISIs). The other three comparisons provided equivocal evidence. Perhaps the findings are partially a result of our methodology. We determined song learning via lyric accuracy, and many participants failed to sing the notes with a high degree of pitch accuracy during the initial learning episode (Fig. 2, top panel). We used degree of pitch accuracy as a secondary measure (absolute value of cents off-pitch), as this measure is less susceptible to less than perfect ability to sing. Again, we found a lack of spacing effect for note learning. It is worth noting that session 1 performance was good, with accuracy high enough that a spacing effect should have been detectable at final test, so production skills alone do not appear to be responsible for our lack of observed spacing effect for notes. We are unable to determine the degree to which note recall is a function of declarative memory versus motor skill. The effect size for complex motor tasks (*d* = 0.11 to *d* = 0.42; Donovan & Radosevich, 1999) is smaller than the effect size for verbal learning (*d* = 0.85; Cepeda et al., 2006). It could be that the effect size of the spacing effect is smaller for note learning than syllable learning; with bimodal data, we are unable to confirm or deny this possibility, since we cannot compute comparable effect sizes. Most of our Bayesian analyses of note recall showed conclusive support for a null effect, so we do not believe insufficient power is responsible for our findings. Further research is needed to understand if the spacing effect is conducive to note learning.

Although there are no spacing studies for tune memory to compare, there is evidence for pitch consistency in long-term tune recall (Halpern, 1989; Schlemmer, 2002; Wallace, 1994). Halpern (1989) found that in a population of musicians and non-musicians without absolute pitch, starting pitches for familiar songs were reproduced with considerable consistency across a 48-h interval. For both spaced conditions, at test 2.1, more than half of the words and notes were forgotten. There was much better note recall in the massed than in the spaced conditions. The evidence is that for a single learning session, note recall after a 10-min gap is accurate; at two days or one week, note recall is inaccurate, with no difference between the two spaced intervals. That note recall of a novel melody is more accurate after 10 min than after two days or one week is consistent with previous research. Long-term accuracy in pitch reproduction has been shown for familiar songs (Halpern, 1989; Rubin et al., 1998), but recognition studies of novel melodies frequently show poor pitch recall (Halpern & Müllensiefen, 2008; Halpern & O’Connor, 2000).

Unlike most of the spacing effect verbal learning literature (Cepeda et al., 2006, 2008, 2009), there was evidence for no spacing effect between 2-day and 1-week gaps. There does not appear to be a strong optimal ISI for lyric recall, which deserves confirmation, using a wider range of ISIs, in a future study. Unlike most of the verbal spacing effect literature, lyrics have strong internal cues and a single theme, whereas most studies have investigated random word pairs, trivia facts, or other sets of discrete items. In essence, the song contained a single cue (the first two notes and words), followed by a series of connected cues (the lyrics, reinforced by the stress patterns and cadences of the melody). Likewise, we see evidence against differential forgetting between 2-day and 1-week gaps, at tests 2.1 and 2.2.

A study of the spacing effect in song necessitates a confound between notes and words in both the learning and retrieval phase. Given this confound between notes and words, it is not clear from this study whether a melody without words and a poem without a melody are influenced by spaced learning. It is clear, however, that memory for words and music together is strongly influenced by spacing, especially when there is a cue that can trigger the ongoing structural constraints in the material. The effectiveness of recall depends on a gap somewhere between 10 min and two days. It is entirely possible that an interval of sleep may be the determining factor, as with other studies of spacing in verbal learning (Bell et al., 2014). More notes were consistently produced than words at the final tests, which may indicate that where words were forgotten, notes were used as a framework to enable subsequent word retrieval. This is consistent with previous research showing the use of the rhythmic and melodic framework of the song as an on-going cuing system for word retrieval (Purnell-Webb & Speelman, 2008; Rubin, 2006; Wallace, 1994; Wallace & Rubin, 1991).

Our study has practical ramifications for singers. Outside the laboratory, performing materials may encompass thousands of words and thousands of notes—far more than the simple two-verse song we used as our stimulus material. We suggest that spaced practice of short segments learned to criterion may be an effective first step toward designing a practice schedule that ensures the greatest possible memory security in performance.

## Conclusions

This study showed that the spacing effect can be used to help memory for song. Unlike most verbal learning studies, we failed to show an inverse-U pattern in which retention improved with increased spacing and then decreased with further increases in ISI length (Cepeda et al., 2008). Future studies should examine whether this result was due to our choice of inter-study intervals, or whether the large number of cues present in songs contributes to better memory from spacing regardless of ISI. In the future, two additional follow-up studies are clearly indicated, examining whether the spacing effect improves memory for tunes without words and for lyric poetry.

## Data Availability

The dataset and materials supporting the conclusions of this article are available in the OSF repository, https://osf.io/mus3c/.

## Appendix 1

See Tables 5 and 6.

**Table 5:** Demographic characteristics of experimental groups

	10-min	2-day	7-day	BF_10_
Age	21.0 (2.5)	21.0 (3.5)	20.9 (3.3)	0.10
Gender	20F/10 M	11F/18 M	14F/14 M	0.98
Guardian years of education	15.0 (3.1)	14.8 (3.5)	15.6 (2.9)	0.15
Bilingual (0 no–10 yes)	5.5 (3.1)	5.4 (3.3)	5.4 (3.9)	0.10
Singer	*n* = 8 no *n* = 22 yes	*n* = 9 no *n* = 20 yes	*n* = 6 no *n* = 22 yes	0.10
Took voice lessons	*n* = 17 no *n* = 13 yes	*n* = 22 no *n* = 7 yes	*n* = 15 no *n* = 13 yes	0.49
Hours per week of singing	6.5 (7.5)	5.3 (4.3)	5.2 (4.7)	0.15
Sheet music reading proficiency (10 = most proficient)	5.5 (2.6)	6.4 (2.7)	6.1 (2.5)	0.23
Log number of songs performed from memory	1.1 (1.0)	1.1 (1.0)	1.4 (0.91)	0.17
Anxiety at testing (10 = extremely anxious)	3.3 (2.7)	3.0 (2.5)	3.1 (2.4)	0.11
PROMS test of music perception ability	22.8 (4.8)	22.5 (3.7)	23.7 (3.9)	0.17

Mean and SD are shown

**Table 6:** Inter-rater reliability (Pearson’s r)

Measure	Test 1.1	Test 1.3	Test 2.1	Test 2.2	Test 2.4	Test 3.1	Test 3.2
Word omissions	NA	1.000	0.999	0.999	0.986	0.999	1.000
Note omissions	NA	1.000	0.999	1.000	0.986	0.999	1.000
No. cents off quarters	1.000	0.999	0.966	0.999	1.000	.899	.888
Quarter length	0.959	0.983	0.783	0.964	0.984	0.951	0.972
Word errors	NA	0.958	0.991	0.922	0.969	0.940	0.924

## Appendix 2

See Tables 7, 8 and 9.

**Table 7:** Learning outcome: syllables (test 1.3), for massed, 2-day, and 1-week ISIs

Measure	Massed *M* (*SD*)	2 day M (*SD*)	1 week M (*SD*)	BF_10_ overall	BF_10_ massed vs. 2 day	BF_10_ massed vs. 1 week	BF_10_ 2 day vs. 1 week
Syllable omissions	0.033 (0.18)	0.069 (0.26)	0.036 (0.19)	0.13	0.31	0.27	0.31
Syllable errors	0.43 (0.65)	0.50 (0.67)	0.63 (1.00)	0.15	0.28	0.37	0.31
Syllables added	0	0	0

**Table 8:** Learning outcome: notes (test 1.3), for massed, 2-day, and 1-week ISIs

Measure	Massed *M* (*SD*)	2 day M (*SD*)	1 week M (*SD*)	BF_10_ overall	BF_10_ Massed vs. 2 day	BF_10_ Massed vs. 1 week	BF_10_ 2 day vs. 1 week
Cents off-pitch, quarter notes	18.7 (36.8)	17.4 (31.7)	9.8 (22.8)	0.17	0.27	0.44	0.42
*SD* of cents off-pitch, quarter notes	31.7 (18.2)	38.5 (18.6)	36.0 (20.4)	0.22	0.62	0.36	0.30
Absolute value of cents off pitch, quarter notes	38.7 (26.3)	38.7 (21.7)	32.4 (12.2)	0.20	0.26	0.46	0.56
Number of quarter notes off-pitch	19.2 (19.8)	19.1 (15.8)	14.2 (8.7)	0.15	0.32	0.32	0.27
Proportion of quarter notes off-pitch	0.27 (0.28)	0.27 (0.22)	0.20 (0.12)	0.22	0.26	0.50	0.63

**Table 9:** Session 3 trials to criterion, for massed, 2-day, and 1-week ISIs

Measure	Massed *M* (*SD*)	2 day M (*SD*)	1 week M (*SD*)	BF_10_ overall	BF_10_ Massed vs. 2 day	BF_10_ Massed vs. 1 week	BF_10_ 2 day vs. 1 week
Session 3 trials to criterion	3.37 (1.45)	2.72 (1.41)	2.46 (1.48)	1.11	0.93	2.62	0.32

## Appendix 3: Study design

**Table:** Session 1: training to criterion

Melody practice	Test 1.1	Song practice	Test 1.2	Test 1.3

**Table:** Inter-study interval (10 min, 2 days, or 1 week) Session 2: relearning

Test 2.1	Test 2.2	Song practice	Test 2.3	Test 2.4

**Table:** Retention interval (3 weeks) Session 3: final test and relearning

Test 3.1	Test 3.2	Relearning to criterion

## Abbreviations

ISI: Inter-study interval
RI: Retention interval
PROMS: Profile of music perception skills
